# Regional forecasting of COVID-19 caseload by non-parametric regression: a VAR epidemiological model

**DOI:** 10.3934/publichealth.2021010

**Published:** 2021-02-01

**Authors:** Aaron C Shang, Kristen E Galow, Gary G Galow

**Affiliations:** 1 University of Oxford Medical Sciences Division; Oxford OX3 9DU, UK; 2 Hackensack Meridian School of Medicine; Nutley, NJ 07110, USA; 3 Direct Energy LP; Iselin, NJ 08830, USA

**Keywords:** novel coronavirus, COVID-19, vector autoregression, VAR, model, prediction, SIR, SARS-CoV-2

## Abstract

**Objectives:**

The COVID-19 pandemic (caused by SARS-CoV-2) has introduced significant challenges for accurate prediction of population morbidity and mortality by traditional variable-based methods of estimation. Challenges to modelling include inadequate viral physiology comprehension and fluctuating definitions of positivity between national-to-international data. This paper proposes that accurate forecasting of COVID-19 caseload may be best preformed non-parametrically, by vector autoregression (VAR) of verifiable data regionally.

**Methods:**

A non-linear VAR model across 7 major demographically representative New York City (NYC) metropolitan region counties was constructed using verifiable daily COVID-19 caseload data March 12–July 23, 2020. Through association of observed case trends with a series of (county-specific) data-driven dynamic interdependencies (lagged values), a systematically non-assumptive approximation of VAR representation for COVID-19 patterns to-date and prospective upcoming trends was produced.

**Results:**

Modified VAR regression of NYC area COVID-19 caseload trends proves highly significant modelling capacity of observed patterns in longitudinal disease incidence (county R^2^ range: 0.9221–0.9751, all p < 0.001). Predictively, VAR regression of daily caseload results at a county-wide level demonstrates considerable short-term forecasting fidelity (p < 0.001 at one-step ahead) with concurrent capacity for longer-term (tested 11-week period) inferences of consistent, reasonable upcoming patterns from latest (model data update) disease epidemiology.

**Conclusions:**

In contrast to macroscopic variable-assumption projections, regionally-founded VAR modelling may substantially improve projection of short-term community disease burden, reduce potential for biostatistical error, as well as better model epidemiological effects resultant from intervention. Predictive VAR extrapolation of existing public health data at an interdependent regional scale may improve accuracy of current pandemic burden prognoses.

## 1. Introduction

The ongoing global outbreak of novel coronavirus disease 19 (COVID-19, caused by the coronavirus strain SARS-CoV-2) represents a leading public health emergency. To date, it has affected at least 217 countries and territories, leading to more than 100 million positive cases and 2.2 million deaths as of January 2021 [1]. In this time, COVID-19 has demonstrated extensive unique properties including an extended incubation period, likelihood for high levels of asymptomatic transmission, and a very nonspecific symptomology leading to difficulty in accurately identifying positive cases. In addition, COVID-19 exhibits a high basic reproduction ratio (R0) combined with a high prevalence of cases having mild clinical presentation. Current literature shows that up to 80% of infected people may exhibit negligible respiratory impact [2]. Subclinically afflicted individuals are more likely to engage with communities during active infection periods and less likely to seek out health care services, given inconspicuous course of illness as well as minimal impact on quality of life. Collectively, these factors inherent to COVID-19 have impeded epidemiologic characterization and contributed marked difficulty towards efforts at accurate prognostication of future disease behaviors.

Many contemporary infectious disease models rely on the S-I-R (susceptibility, infection, removed or resistant) population transmission-based framework. However, this approach, which utilizes a multitude of assumed variables and draws data from highly divergent sources, has received prominent criticism over predictive inaccuracies skewing both positive and negative in trend [3],[4]. In combination with computational challenges associable to estimating the atypical real-time development of COVID-19, significant concerns have been raised regarding data integrity when collected at a macroscopic level, both in terms of accuracy for reported figures [5] between contrasting sources (i.e. separate agencies) as well as differing national-to-international classifications for what constitutes a positive COVID-19 identification [6]. More involved attempts to model the projected influence of changing population dynamics as well as quarantine measures, as operationalized through SUQC (susceptible, unquarantined infected, quarantined infected, confirmed infected) models, have likewise noted significant variation in data veracity, which has reduced predictive value even within a weekly timeframe [7],[8]. Therefore, it is the position of this paper that accurate prediction modelling of COVID-19 disease progression at a community level in an upcoming weeks-to-month timeframe is best achieved at a local rather than macroscopic level, be built upon reliable data sources of observed COVID-19 patterns, and be adaptable to fluctuating inputs that change with management, contextualizing that pandemic processes will likely occur non-linearly and with imperfect (and difficult to express via singular-entity variables) causal relationships.

## 2. Methods

Herein a modified vector autoregression (VAR) model based upon daily U.S. county-level public health agency test-positivity rate and mortality data in COVID-19 for the NYC metropolitan area (operationalized through selected diverse, representative counties) is developed with prediction of upcoming regional disease patterns given late July 2020 disease control status as well comprehensive data encompassing past local caseload patterns (March 8 to July 23, 2020). VAR modelling attempts to quantify interrelationships wherein two or more time-dependent series are collectively impactful upon observable trends, wherein all referenced variables are treated as being endogenous (*y*, dependent), rather than necessitating fundamental independent (*x*) assumptions, as might be appreciated in S-I-R projections. Reliable county level COVID-19 morbidity data was derived from governmental sources in the New York metropolitan area comprising of seven designated major counties within New York State (NYS) of noted COVID-19 burden (New York, Bronx, Rockland, Nassau, Queens, Westchester, Kings respectively; specific county data inputs available as supplemental material) and exported to classification on a daily continuum. Per protocol vector autoregression (VAR) of the filtered dataset was then conducted, sequentially in seven repetitions utilizing each individual county progression as the dependent (*y*) variable. Subsequently, one through three day lagged values were used as the independent variable matrix inside the encompassing VAR framework.

VAR modelling for each county was identified using a backward stepwise regression approach, applied to identify the final regression equation using a validated Akaike Information Criteria (AIC) methodology. AIC represents an estimator of out-of-sample prediction error that quantifies the amount of information lost by using the model to approximate an underlying series (the true daily COVID-19 new caseload). That is, it signifies juxtaposition of the error created by using a specific model against the actual data. The lesser the degree of information loss therefore, the better a trialed model is valued. AIC was used in this manner to sort through and quality-control the entirety of possible county-specific VAR models, relative to each of all alternative models under logical consideration. This process was done in the R project software, using the &ldquo;olsrr: Tools for Building OLS Regression Models” package for procedures. As per the cited backward regression methodology, model analysis started with the full matrix of all lagged variables and then an AIC value was calculated for each variable. Variables that did not meet AIC criteria were iteratively dropped, until a final set of perceived best-representative regression variables, that all met AIC criterion, for county-wide COVID-19 caseload projections resulted. As reference, the initial equation for New York County is shown below.

The equation in general form (variables interchangeable by individual county):

$$NY_{t}=\alpha +\overset{3}{\underset{j=1}{\sum }}\beta 1_{j}B_{t-j}+\overset{3}{\underset{l=1}{\sum }}\beta 2_{l}K_{t-l}+\overset{3}{\underset{m=1}{\sum }}\beta 3_{m}N_{t-m}+\overset{3}{\underset{n=1}{\sum }}\beta 4_{n}NY_{t-n}+\overset{3}{\underset{p=1}{\sum }}\beta 5_{p}Q_{t-p}+\overset{3}{\underset{s=1}{\sum }}\beta 6_{s}R_{t-s}+\overset{3}{\underset{u=1}{\sum }}\beta 7_{s}W_{t-s}+\varepsilon_{t}$$ (1)

wherein:

*α* = Intercept

*β*1, *β*2, *β*3, *β*4, *β*5, *β*6, *β*7 = Regression coeffients

*B* = Bronx County COVID cases

*K* = Kings County COVID cases

*N* = Nassau County COVID cases

*NY* = New York County COVID cases

*Q* = Queens County COVID cases

*R* = Rockland County COVID cases

*W* = Westchester County COVID cases

*ε*_t_ = Regression error term

*t* = Time subscript

*j*, *l*, *m*, *n*, *p*, *s*, *u* = individual counting subscripts for lags

A representative implementation of output model selection for New York County follows:

$$<NY_{t}>=1.14716+<0.15238B_{t-1}>-<0.26944K_{t-2}>+<0.19612N_{t-1}>+<0.53047NY_{t-1}>+<0.17727NY_{t-3}>-<0.14725Q_{t-2}>+<0.15276R_{t-1}>-<0.10518R_{t-2}>+<0.24493W_{t-2}>-<0.10089W_{t-3}>$$

wherein variables are as defined previously, and < x_*t*_ > represents the expected value operator for new COVID cases in the county of reference (here New York county) at time *t*.

The described AIC analysis sequence was stopped when the backward process indicated that an optimal solution (model) had been reached, in that the latest attained AIC value (minimization of regression variance) exists less than all other possible candidate values. Translationally, the determined endpoint therefore indicates statistically that remaining independent variables correspond to the best predictors of disease progression for the input county data per the criterion. Modelling was performed by log of cases rather than raw caseloads, followed by translation into raw daily future caseload projections. This decision derives from the fact that in tracking available COVID-19 data historically, the distribution of new cases generally followed a lognormal distribution more closely than it did a normal distribution. All resultant equations were utilized to forecast predicted disease progression in the 7 counties (AIC-selected county regression models, available as supplemental data). County one-day and multi-day ahead forecasts of daily caseloads were generated from best-fit VAR models.

**Figure 1.**
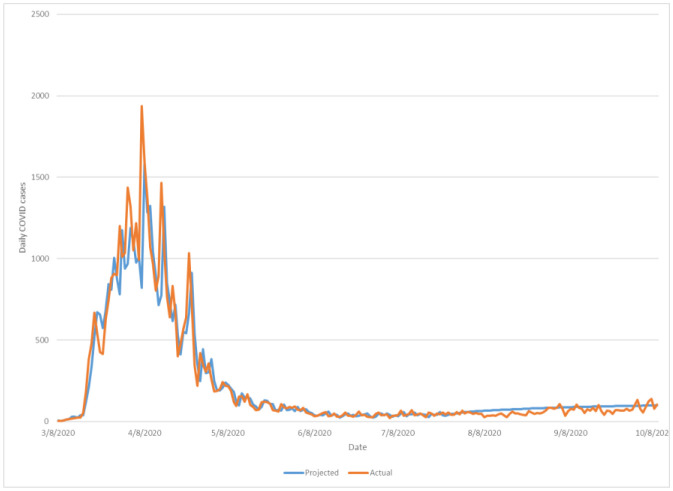
Multi-step (11 weeks) ahead comparison of projected upcoming daily COVID-19 caseloads against observed new daily cases of Nassau county-wide COVID+.

**Figure 2.**
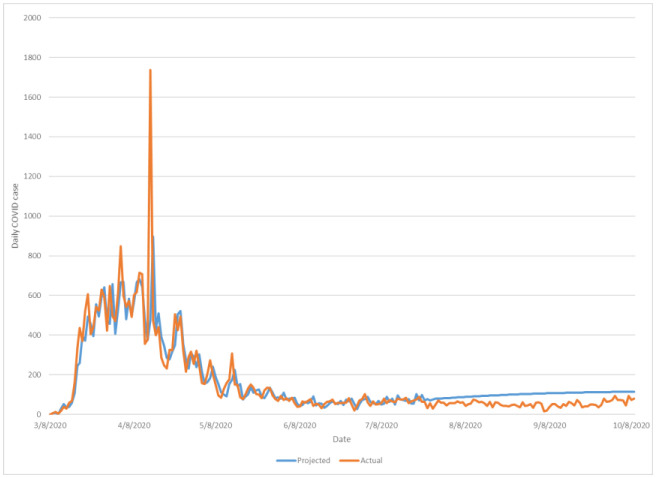
Multi-step (11 weeks) ahead comparison of projected upcoming daily COVID-19 caseloads against observed new daily cases of New York county-wide COVID+.

Statistical determination of strength of prediction and association between VAR model projected values to observed quantities was the primary outcome of analysis in the present study. A one-step ahead projection was first examined to quantify goodness-of-fit for the VAR model to represent existing COVID trends. To establish longer-term prediction power for this model, an approximately 11-week (from latest date of model adjustment on July 23 forecasting to October 9, 2020) simulation was performed following for all 7 incorporated study counties (sample New York and Nassau County projections demonstrated in Figures 1–2, counterpart projections for other counties and comprehensive raw projection outputs available as supplemental Figures 3–6). Following evaluation of derivative findings, critical examination of model implications alongside relevant strengths and limitations was offered (see Discussion).

## 3. Results

Correlation between predicted and observed COVID-19 new case values proved consistently significant at one-day-ahead predictions given latest daily caseload data (p < 0.001 all counties between March 8, 2020 to July 23, 2020), indicative of a high degree of model accountability for the multifactorial influences which have moderated COVID-19 caseload changes from the onset of pandemic spread (and data availability) to present. By assessment therefore of both past caseload and present disease status representation, the AIC-driven modified VAR model described presently was deemed appropriately representative of disease evolutions to-date and by extension thusly appropriate for extrapolation of future COVID-19 potentiality.

Having established model fidelity (based upon daily COVID-19 new case reports thru July 23, 2020 across the included jurisdiction of interest), wider prediction of caseloads beyond one-step ahead assessment was performed, likewise independently for each county dataset. Herein estimates on daily new COVID+ case incidence was generalized to 11 weeks into the future (to October 9, 2020), again utilizing the latest modified VAR regression per-county based on July 23, 2020 input figures. Examination of this model's expectations for disease progression across a 10 to 11 week (from July 23, 2020 onwards) for the greater NYC region revealed relatively consistent, gradual increases in COVID-19 prevalence across all seven counties of interest. Average percentage of predicted daily case growth from July 23 to October 9, 2020 ranged from approximately 75% to 135% between counties, a significant although less distinct acceleration as compared to numerous locales nationally or periods of rapid disease transmission in the same NYC metro region during preceding months of 2020. Demonstration of county-specific projected versus observed COVID caseloads for Nassau County and New York county (Manhattan) are demonstrated in Figures 1 and 2 respectively, wherein the modified VAR projection was able to relatively consistently demonstrate expected new daily case quantities at one-step-ahead based on existing data influence on the county-specific models throughout the duration of the 11-week ahead period of prediction; all other county projections are included in supplemental Figures 3–6.

As depicted in Figure 1 (Nassau sample), based on COVID-19 containment status and case progression in Nassau County (and similarly across all seven examined counties in this study) from the onset of COVID community spread in early March through late July, the VAR projected rate of future caseload anticipated relatively stable, small-amplitude increases across the upcoming 11-week predictive timeframe. By illustration, in contrast to the lowest daily new cases of 26 persons noted June 28, 2020 (smallest quantity recorded since March 12, 2020, during the earliest stages of community COVID-19 outbreak in the NYC region), a proposed VAR model for Nassau County suggested that by October 9, 2020 daily caseload would likely rebound to approximately 105 or more positive cases per day. Upon retrospective comparison of the actual recorded COVID+ daily values to those projected, strength of predictive correlation remained high, with error rate of new daily test positives in Nassau county minimized at an average of roughly 22 patients for the entirety of the 11-week prediction period from July 23 to October 9, 2020 (Table 1). This measured uncertainty also did not increase throughout the timeframe of projection (as dates of estimation became further in future from time of modelling).

Similar projective extrapolation as modelled by the VAR function upon 7/23/2020 caseload data for New York county is demonstrated in Figure 2, and all other county models are shown in supplemental materials as aforementioned. New York County VAR-estimated to actual daily caseloads exhibited a comparable average error of approximately 46 patients per day countywide, comparable to both the Nassau findings described previously as well as all other county samples (error range of 20 to 90 patients per county per day; see Table 1). For all analyzed counties in this study furthermore, the out-of-sample (11-weeks prediction) error from subsequently observed was similar if slightly smaller than in-sample (VAR representation of past and current data used for projection, 3/8–7/23/2020) which intuitively suggests that at an 11-week frame of projection the current VAR model for the 7 presently selected NYC metropolitan counties was able to accurately correlate future forecasts with existing community data.

**Table 1.** Multi-step predictive VAR model mean absolute error (MAE) of daily COVID-19 cases across select NYC metro counties.

MAE	Bronx	Kings	Nassau	New York	Queens	Rockland	Westchester
In Sample	72.04	96.10	65.78	48.03	109.27	31.86	62.03
Out of Sample	67.72	71.17	21.81	46.36	88.79	23.54	34.36
Total	70.48	87.10	49.90	47.43	101.87	28.86	52.04

Table 1 Mean absolute error of daily predicted COVID-19 positive caseloads per county by modified VAR regression. Note error levels are separated by in-sample (VAR representation of past and current case trends through 7/23/2020) and out-of-sample (future projections from 7/23–10/09/2020). MAE units of measurement as demonstrated is individual patients (persons).

Of note, wherein the sampled NYC region data over the period of VAR analysis has relatively reliably progressed within relatively small magnitudes of caseload variation, the capacity for this VAR model's “shock”-predicting capacity (commonly utilized in econometrics to predict market response to individual events) relative to exponential real-time case increases as in Florida or Texas, could not be efficaciously evaluated. A new case data outlier recorded on April 14, 2020 (1737 cases) resulting from inclusion of previously uncounted presumed COVID positives (owing to changes to data classification guidelines by the CDC and NY State Department of Health) provides only a very limited amount of insight towards this consideration given the limited sample size (including one-day duration) and extenuating data spike circumstances (case allocation reclassification). With wider available data and empirical course of COVID-19 community transmission however, the strong expectation persists that adapted VAR impulse-response evolution estimates may well concurrently provide a moderately accurate accommodation of periods of either rapid growth or decay in regional COVID-19 new identifications incidence. Empirical quantification of VAR model adjustment from extreme trends of epidemiological data alteration therefore requires further investigation and follow-up.

## 4. Discussion

This paper proposes that COVID-19 community containment and modeling may be best represented at a local or regional level, given persistent uncertainties regarding data quality and reliability at national and global scale. Furthermore, the study develops a modified VAR-based predictive representation based on New York City metropolitan area (7 NYS counties) disease data from July-through-October at the county level, in order to demonstrate prospective near- and medium-term epidemiological trends for COVID-19 cases. Note that the presently proposed model has been built with a minimum of distributional assumptions (inferences on either variable interrelationships or resultant caseload outcomes), allowing any results to closely mirror observable empiric trends [9]. Moreover, a VAR foundation is useful in examining the dynamic effects of each variable on all others simultaneously [10]. Across incorporated datasets across major NYC regions subjected to VAR analysis, the modified VAR model suggested that under late July 2020 containment status and continued maintenance of local management measures (data available to the VAR system at the time of predictive modelling) COVID-19 cases would expect to rebound in daily incidence, although at a less marked (or unsustainable) pattern as seen in numerous other regions of the United States. Through comparison of actually recorded subsequent caseload figures over the NYC metropolitan region over a following 11-week period (until October 9, 2020), the accuracy of VAR projections at caseloads at such a projective timeframe proved generally accurate (Figures 1–2) with average error in expected versus actual daily COVID cases falling approximately between 20 to 90 patients per county per day (Table 1, out-of-sample error). Considering that U.S. management strategy of COVID-19 has centered around reduction of peak disease burden to a systemically manageable level rather than systematic viral eradication [11], evidence proposed presently would suggest that the greater NYC region remains amongst few counterparts currently close to meeting such expectations of temporally-dispersed and manageable COVID case burdens upon local healthcare infrastructure. However, as has been observed across both the U.S. as well as numerous countries globally, changing community containment stringency as well as seasonal population patterns may considerably exacerbate case progression beyond what has been projected by this model herein.

A modified VAR model carries marked regression capacity of representing observed caseload patterns alongside generally significant predictive value for estimation of new COVID-19 cases as shown by an analysis of NYC metropolitan county data from the period of July 23 (latest date of data entry and by association model adjustment) to October 9, 2020. This functionality and efficacy denotes a significant improvement upon both prospective and retrospective accuracy reached by numerous mainstream state- and national-level models founded upon variations of S-I-R epidemiologic interactions [12],[13], and as such may be utilized to better prepare regional public health and medical resources for expected immediate-term changes to new case patterns. In context of this model's mean daily COVID caseload error of between 50 to 100 individuals per county, this variability in the authors' opinion represents a practically manageable level of uncertainty at the county scale (unlikely to cause sudden, unexpected medical resource strain).

Furthermore, given literature which has noted that a major challenge to accurate prediction of pandemic modeling at a macroscopic level derives at least partially from inherent differences between diverse populations [14],[15], a past-data driven model such as that currently described may prove more applicable to individualized regional disease patterns in terms of predicting upcoming development since it amalgamates rather than reduces influential epidemiological influences impactful upon overall observed disease patterns. Concurrently, wherein across S-I-R models multiple assumptions—essentially independent predictions—have to be made before dependent caseload projections can be obtained, relative to VAR only observed relevant data patterns have the capacity to influence the resultant model's expectations and implications. Moreover, wherein S-I-R type models require at a minimum distinct representations of three separate patient population subclasses, more complex models (i.e. SUQC) predicate even more indirectly upon further assumptions of quarantined patients, mortality, or any number of further uncertain disease properties. By contrast, the present VAR modality requires only empirical caseload data as input, and directly translates this single independent dataset towards forecasting of upcoming new cases; additional error from multiple necessary assumptions or prospective confounding variables is minimized in this VAR implementation. The authors therefore propose that singular estimation of COVID-19 cases represents an appropriate application for the described model, given dynamism in disease severity and prevalence across socio-demographic boundaries [16],[17] difficult to linearly represent through susceptibility variable valuation.

### 4.1. Public health applications and outlook of VAR-based modelling

S-I-R models are fundamentally parametric and so implicitly assume an S-shaped curve for the life of an outbreak. As shown by previous data from representative NYC metropolitan counties however, observed daily infection counts did not in fact follow a logarithmic or logistic sigmoid regression. Instead, new infections started slowly and grew exponentially (as would be expected with a traditional S-I-R model) but consequent infection quantities declined significantly less precipitously and in a more linear fashion than would be projected by many transmission-variable derived models [18]. Prominently propagated examples of S-I-R constraints in active pandemic modelling can be viewed through the Imperial College London (U.K.) and University of Washington (U.S.) morbidity and mortality projections, each of which demonstrated major inflexibility to changing containment conditions and increased data availability for COVID transmission rates and necessitated frequent (bi-weekly respective to the U.S. model) major corrections to upcoming caseload predictions [19]. At both extremes of S-I-R projection from the Washington U.S. model, total COVID morbidity in the U.S. by late October 2020 was variously estimated in May and July as 4 million and 20 million with significant variability in between. To correct for theorized S-I-R methodologic limitations, this paper utilizes a modified multiple regression analysis to forecast county-by-county (Bronx, Kings, Nassau, New York, Queens, Rockland, and Westchester counties) outbreaks in NYC through autoregressive lagged values as well as lagged values from the counterpart neighboring counties. VAR adaptation in this manner allows the produced model to account for the fact that regional viral dissemination encompasses a function of past cases within individual counties as well as established shifts with neighboring locales, given inevitable dynamism of population movement and disease spread across a macroscopically interconnected geography.

The largely non-parametric COVID projection model discussed in this study is founded upon practices inherent to traditional vector autoregression (VAR) modelling within the field of econometrics. VAR models have been well validated in economic studies wherein intertemporal relationships between variables are hypothesized, a characteristic which implies considerable utility in medicine. In the setting of novel infectious diseases, adaptation of VAR allows for the simultaneous evolution of multiple interconnected yet singularly unquantifiable disease-modifying variables such as disease basic reproduction number (R_0_), test availability, asymptomatic transmission and population susceptibility status or degree of social contact over time. It is the position of this paper therefore, that a VAR-based modelling of regional disease patterns carries highly translatable advantages in COVID-19 modelling derived from this described allowance for multiple evolving variables. Unlike S-I-R or univariate mechanisms, VAR is not critically driven by assumptions or awareness of the underlying forces that impact variable behavior or patterns. VAR as mentioned represents a nonparametric model, indicating that there is no pre-existing shape assumption made about the disease-progression curve; instead, any visualized curve shape is generated solely based on trends in pre-existing data. This contrasts with the parametric S-I-R model that assumes an S-shaped curve. All predictions are based only specific to the ongoing development of COVID-19 community disease spreads, this quality is critical given that all derivative predictions by VAR are extrapolated from existing variable trends (derived from daily case rate) rather than theorized structural statistical relationships.

### 4.2. Model limitations

Major constraints in predictive value by this model persist mainly from quality of input data. Considering ongoing uncertainties as to the protective value of COVID-19 antibodies in terms of both efficacy as well as duration of immunity [20],[21], unaccounted for variability is introduced through the possibility of a changing susceptible baseline population longitudinally. Furthermore, it remains very difficult to regularly predict upcoming disease patterns based on past data for timeframes greater than a few weeks-to-months in advance given highly volatile quarantine and social distancing measures across most surveyed locations. Whereas the improved reliability for predicting caseload (dependent) outcome shifts given a mature data cycle of COVID-19 outbreak as well as management in the NYC region should improve forecast accuracy as opposed to manual estimation of R_0_ or further variables, it remains largely impossible to quantify with any confidence what impact longitudinal dynamisms such as virus mutation or previous exposure may introduce. When considering COVID-19 represents arguably the most extensive and bio-statistically confounding global pandemic since the 1918 influenza outbreak, there persists high likelihood that the most productive analysis and shaping of future disease modeling practices will occur at the conclusion of generalized COVID-19 spread, wherein detailed examination of disease predictions via contemporary modelling methodologies may be objectively contrasted against quantifiable longitudinal outcomes.

Viewed from a practical perspective moreover, an inherent limitation to VAR-derived epidemiological modelling lies in the lagging of observed-to-predictive patterns, particularly relevant compromise in the setting of large, sudden data modifications. Specific to COVID-19 and disease dynamics, this is important as salient epidemiologic events such as mass holiday travel or fluctuation viral mutants of differing infectivity variably exert major impacts upon observable downstream caseloads. By illustration, the need for the model to better adjust future projection expectations at a lagging interval to new system shocks (significant deviations) can be preliminarily observed through the model for Rockland County (Supplemental Figure 6), wherein a period of anticipated gradual COVID-case rise (consistent with trends in the encompassing wider region) has been superseded by a rapid early-October week-long spike in cases. In this instance, the predictive VAR model based on 07/23/2020 expectedly becomes more conservative than reality, and a more accurate VAR revision of projections based upon all caseloads through 10/09/2020 would require trend-establishing data of at least several days in order to modify predicted COVID progression with any degree of validity. One potential solution to this inherent VAR lag limitation could be to independently simulate (by VAR) the past influence of such systemic shocks upon subsequently necessary model adjustments, such that newly introduced large data transformations might be able to be accurately accounted for across future instances with only a minimal period of requisite data input (leading to a more adaptable, dynamic model). As discussed previously however, in the setting of the ongoing COVID-19 crisis and associated uncertainty of disease behaviors (see Results), for the present scenario it appears reasonable that only retrospective analysis of extensive datasets in latter stages of the pandemic would enable such a concurrent VAR error-accounting incorporation.

Prognostic modelling involving mortality projections, a similar measure of interest across pandemic progressions, prove more difficult to quantitatively predict given an even greater quantity of uncertainties than is seen in caseload forecasts. Numerous mainstream S-I-R based models have come under heavy criticism for significant temporal adjustments to expected COVID-19 death toll from over the course of the pandemic progression [22],[23], yet meaningful improvement of accuracy in this regard necessitate fundamental shifts in the method and means of epidemiologic disease tracking rather than simple refinement of modelling methodology. A comparable past-trend driven estimate for upcoming mortality rates from the present morbidity model remains unfeasible given wide-ranging uncertainties in vital influencing factors such as incomplete hospital censuses, severity symptomology within hospitalized patients, and a host of incompletely understood genetic-demographic factors which appear to critically moderate COVID-19 prognosis. Perhaps even more articulated in mortality VAR estimates would remain input data integrity, given wildly varying debates in society at present regarding what qualifies as a COVID-19 death as well as noted inconsistencies in timely recording of critical mortality figures. Relative to cumulative COVID-19 deaths and longitudinal incidence then, at present an assumptive variable-driver S-I-R (and derivatives) model would appear the most manageable means for estimation. However, whereas the high variability of input assumptive variables central to this approach was previously discussed in context of caseload projections, herein further uncertainty for more complex SUQC model population subclasses concurrently arises.

### 4.3. Future directions for VAR-based epidemiology

Future directions of development from the currently constructed model may center about using VAR to examine possible delivery mechanism between geographic areas at a wider (i.e. national level), possibly allowing for a more concise predictability regarding warning indicators or at-risk medico-societal practices influencing disease distribution. By example, we might feasibly gain insight towards a potential returned period of caseload maximization in New York City through VAR analysis of lagged-indicators between hypothesized influential demographics (i.e. community socio-ethnic status) or bridging of the urban-rural divide (leading time from urban case development to corresponding rural disease increases) in ongoing hotspots (i.e. metropolitan areas within Florida or Texas as of July 2020). A vigilant, regionally-based monitoring program could therefore quickly identify and potential concerns for community COVID-19 exacerbation or recurrence. Further study of VAR translational value for COVID-19 disease modelling thusly persists in need of model predictive power evaluation between the regional geographic scale illustrated herein, and more macroscopic VAR projections in example at the national level. The upcoming question of public health utility should center on if given robust historical case data, whether VAR-type epidemiologic models will be able to accurately predict population disease progression patterns across larger and more diverse patient or dataset characterizations.

Mathematically, one-to-three days lags of the model variables (relatively short) were chosen with intent to most accurately represent regional disease propagation patterns on a short-term basis, due to a continued state of incomplete understanding regarding COVID-19 and its epidemiologic behaviors. Considering the strong capacity of VAR-founded modelling to account for NYC metro region COVID-19 caseload trends, an appropriate next step may involve trialing of longer lag times (i.e. 1–2 weeks) in order to more efficaciously evaluate extended-period (multiple months) predictive power of this VAR model. In contrast to S-I-R projections wherein predictive uncertainty rises exponentially with increasingly long-term projection, a validated VAR forecast might reasonably be expected to minimize degree of probable variability given that constituent long-term outlooks are based on similarly extended lag-values (extensive past COVID-19 case patterns) rather than parametric extrapolation of static variables made at a singular timepoint.

## 5. Conclusions

According to extensively updated reviews, a significant proportion of previous and ongoing modeling efforts related to COVID-19 are significantly constrained by poor input data quality and inflexible guiding parameters. The current study develops a county-based VAR model derivative from infection data reported in the greater NYC metropolitan area, which demonstrates significantly improved correlation between projected and observed new cases, with promising predictive value at the short (days-to-weeks) to intermediate-term (months) regional level. Furthermore, as described previously the data-driven VAR approach as adapted in this framework remains of greater statistical reliability than assumed dynamics in S-I-R projections, given that all derived inferences built upon the significant quantity of COVID-19 progression data at a local level to-date; this functionality allows for greater tailoring to alternative projections in counterpart geographies or disease settings whilst minimizing quantity of necessary assumptions as long as there exists a robust source of input raw COVID-19 caseload measurements. In example, wherein major increases for New York and comparable U.S. metropolitan areas were separated by significant temporal distribution (March and April versus current June and July respectively), significant similarities in affected population [24] and epidemiologic patterns [25] have been noted between the regions. It would therefore stand to reason that the present modified VAR regression of the initial NYC outbreak of COVID-19 may be not only prove highly applicable to any consequent recurrence in the same geography, but also adaptable to many alternative regions of interest since the currently implemented basis of projection and AIC selection is wholly founded upon disease data available via public records.

It has been well reported in literature that models founded upon estimations of population status relative to disease require significant extrapolation of disease behaviors and epidemiological statistics which may not be appropriate for poorly understood emergent pandemics. Via construction of an adapted VAR extrapolative model which accounts for past patterns of COVID-19 disease trends instead of theoretical representations of pandemic spreading, this paper proposes that existing data-driven estimations of viral dissemination carry significant utility for the real-time projection of regional disease futures. Relative to the ongoing escalation of COVID-19 caseload in many U.S. states, this model was able to predict the gradual increase in COVID cases in the NYC metropolitan region with relative accuracy for a projective period of up to 11-weeks, a highly practical utility especially for information of healthcare infrastructure readiness as well as local public policy related to continued disease containment goals.
