# Supplementary material for: Regional forecasting of COVID-19 caseload by non-parametric regression: a VAR epidemiological model
Source: AIMS Public Health. 2021 Feb 1;8(1):124–36. doi: 10.3934/publichealth.2021010 (PMC7870378; doi:10.3934/publichealth.2021010)
Supplement: Supplementary file 1 [file publichealth-08-01-010-s001.pdf]

AIMS Public Health

AIMS Public Health, 8(1): 124–136.
DOI: 10.3934/publichealth.2021010
Received: 14 November 2020
Accepted: 28 January 2021
Published: 01 February 2021

http://www.aimspress.com/journal/aimsph

Research article

Regional forecasting of COVID-19 caseload by non-parametric regression: a VAR epidemiological model

Aaron C Shang[1,2,*], Kristen E Galow[2] and Gary G Galow[3]

[1] University of Oxford Medical Sciences Division; Oxford OX3 9DU, UK
[2] Hackensack Meridian School of Medicine; Nutley, NJ 07110, USA
[3] Direct Energy LP; Iselin, NJ 08830, USA

* **Correspondence:** Email: aaron.shang@kellogg.ox.ac.uk; Tel: (+1) 410-790-5630.

Supplementary

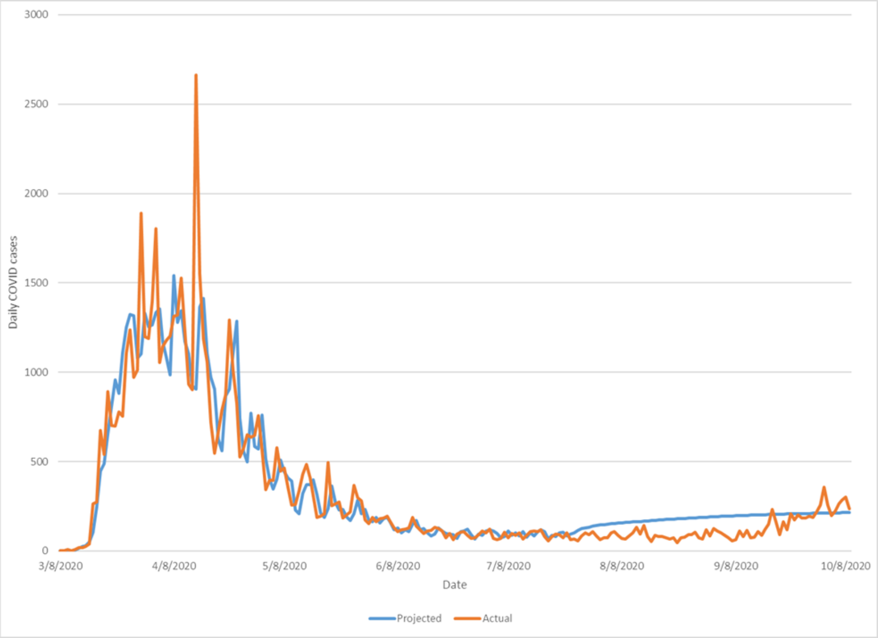

Supplemental Figure 3. Multi-step (11 weeks) ahead comparison of projected upcoming daily COVID-19 caseloads against observed new daily cases of Kings County-wide COVID+.

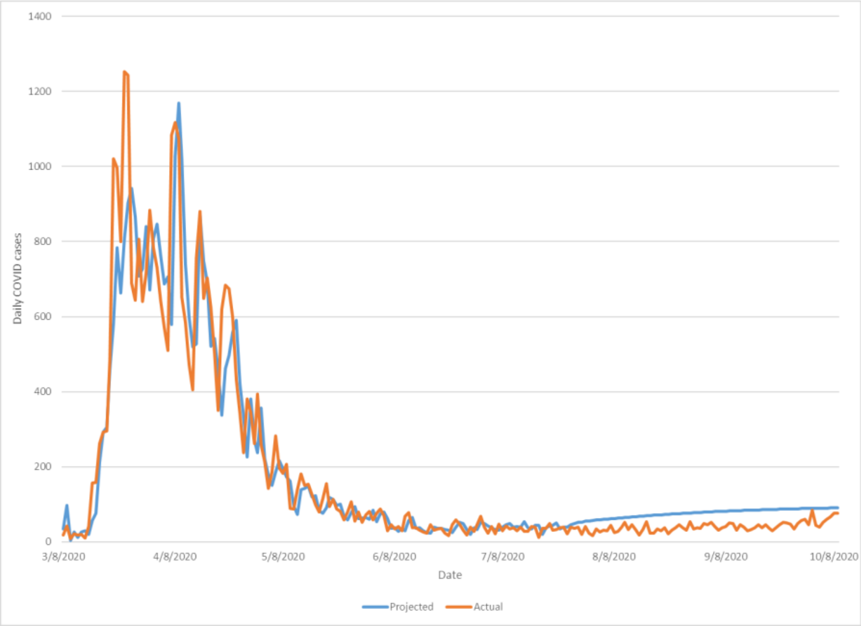

Supplemental Figure 4. Multi-step (11 weeks) ahead comparison of projected upcoming daily COVID-19 caseloads against observed new daily cases of Westchester County-wide COVID+.

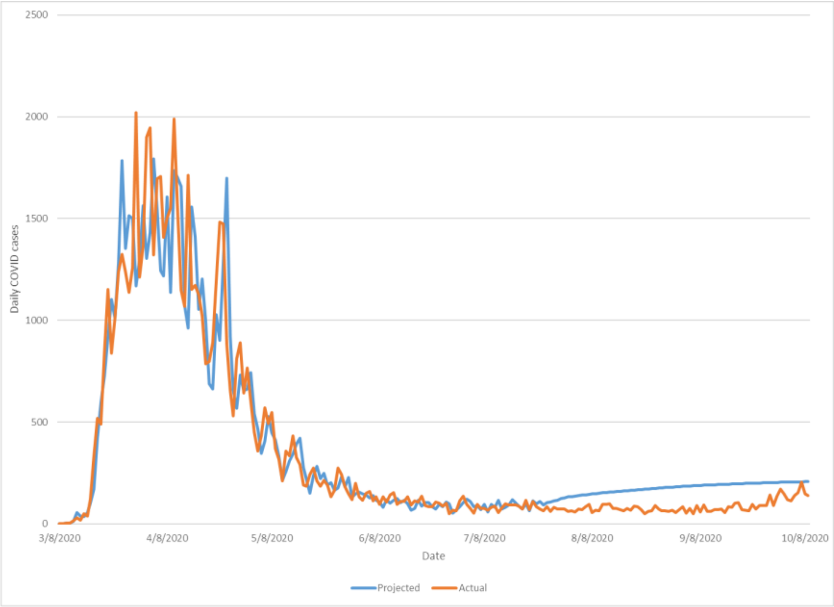

Supplemental Figure 5. Multi-step (11 weeks) ahead comparison of projected upcoming daily COVID-19 caseloads against observed new daily cases of Queens County-wide COVID+.

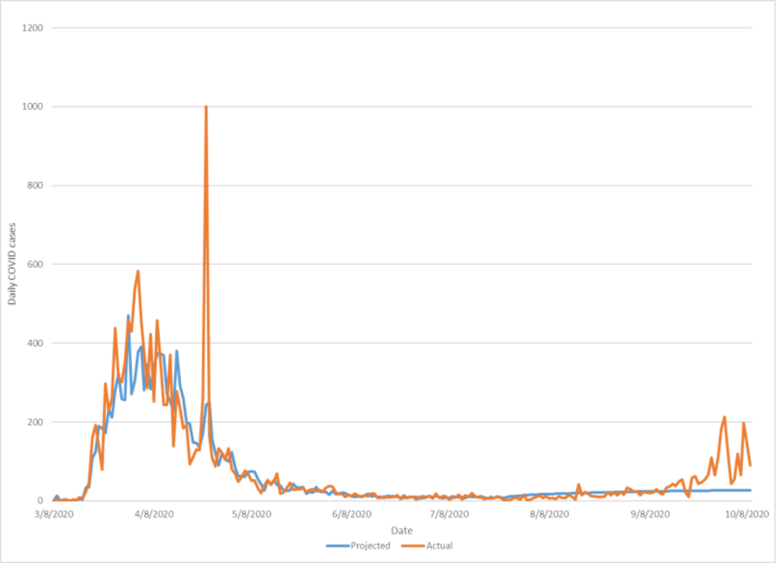

Supplemental Figure 6. Multi-step (11 weeks) ahead comparison of projected upcoming daily COVID-19 caseloads against observed new daily cases of Rockland County-wide COVID+.

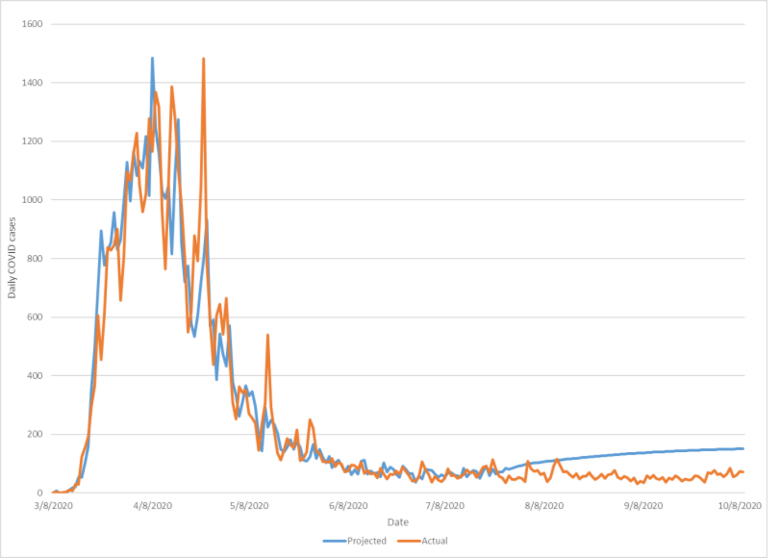

Supplemental Figure 7. Multi-step (11 weeks) ahead comparison of projected upcoming daily COVID-19 caseloads against observed new daily cases of Bronx County-wide COVID+.

AIMS Press

© 2021 the Author(s), licensee AIMS Press. This is an open access article distributed under the terms of the Creative Commons Attribution License (http://creativecommons.org/licenses/by/4.0)
